# *Edwardsiella tarda*, a rare human pathogen isolated from a perihepatic abscess: Implications of transient versus long term colonization of the gastrointestinal tract

**DOI:** 10.1016/j.idcr.2021.e01283

**Published:** 2021-09-06

**Authors:** K. Pham, Y. Wu, G. Turett, N. Prasad, L. Yung, G.D. Rodriguez, S. Segal-Maurer, C. Urban, J. Yoon

**Affiliations:** a Department of Medicine, NewYork-Presbyterian Queens, 56-45 Main Street, Flushing, NY 11355, USA; b The Dr. James J. Rahal, Jr. Division of Infectious Diseases, NewYork-Presbyterian Queens, 56-45 Main Street, Flushing, NY 11355, USA; c Weill Cornell Medicine, Cornell University, NY 10065, USA; d Columbia University School of Nursing, Columbia University, NY 10032, USA

**Keywords:** *Edwardsiella tarda*, Perihepatic abscess, Transient versus long-term colonization

## Abstract

Although gastroenteritis is the most commonly described manifestation of *Edwardsiella tarda* infection, the pathogenesis and transient or long-term colonization of the gastrointestinal tract of this organism in human disease is not clear. We describe a rare manifestation of *E. tarda* infection in a perihepatic abscess in the setting of a patient with perforated cholecystitis and its successful eradication following antibiotic treatment.

## Introduction

*Edwardsiella tarda* is a relatively rare human pathogen that is commonly found in aquatic environments and has been described as an important pathogen in freshwater and marine wildlife [1], [2]. Although gastroenteritis is the most commonly described manifestation of *E. tarda* infection, extraintestinal diseases such as bacteremia, meningitis, cholecystitis, cholangitis, and peritonitis have been reported [1], [2], [3], [4], [5], [6], [7], [8]. In addition, there is an increasing prevalence of *E. tarda* isolated from immunocompromised patients [1], [3]. A major virulence trait associated with this bacterium is its ability to survive and replicate in phagocytes which can lead to transient or long-term colonization of the gastrointestinal tract. However, the precise role of this and additional virulence factors of this organism in human disease is not clear [2]. We describe a rare case of *E. tarda* infection in a perihepatic abscess in a patient with perforated cholecystitis and its successful eradication following antibiotic treatment.

## Case report

An 85-year-old male was admitted to our hospital with five days of fever, hypotension, and right upper quadrant (RUQ) abdominal pain. He had a history of chronic kidney disease (CKD) receiving hemodialysis, hypertension, coronary artery disease, myelodysplastic syndrome, and chronic cholecystitis (medically managed due to extensive comorbid conditions). He also had a history of choledocholithiasis requiring sphincterotomy, stone removal, and ductal dilation. Endoscopic retrograde cholangiopancreatography (ERCP) was performed twice, approximately one year prior to presentation in June 2019 for stone removal and ductal dilation, and in December 2019 for stent removal.

Upon arrival to the emergency department, his temperature was 39.2 °C, blood pressure 139/80 mmHg, pulse rate 108 beats/min, respiratory rate 14 breaths/min, oxygen saturation 95% on room air. The patient was alert and found to have icteric sclerae, but no rash or lymphadenopathy. His lungs were clear to auscultation and heart sounds were regular. His abdomen was soft, but he had mild RUQ abdominal tenderness without abdominal distension. The laboratory data showed leukocytes 5.2 K/µL with 87.8% neutrophils, lactate 2.9 mmol/L, serum creatinine 4.11 mg/dL (unchanged from his last known baseline one month prior in the setting of CKD), serum aspartate aminotransferase 45 U/L, serum alanine aminotransferase 38 U/L, alkaline phosphatase 286 U/L, total bilirubin 1.5 mg/dL with direct bilirubin 0.8 mg/dL (elevated from baseline normal liver function tests done one month prior), lactate hydrogenase 242 U/L, and platelet 85 K/µL (decreased compared to baseline normal one month prior).

Abdominal ultrasound demonstrated a 2.7 cm gallstone impacted at the gallbladder neck.

Contrast enhanced computed tomography (CT) demonstrated a new perforated cholecystitis with perihepatic abscess and mild biliary dilation with pneumobilia suggestive of underlying bilioenteric fistula, which were new compared to prior imaging done one year prior.

Empiric antibiotic treatment with intravenous piperacillin-tazobactam and vancomycin was administered after blood cultures were obtained. A drainage catheter was placed by interventional radiology for drainage of the biliary and perihepatic abscesses. Abscess cultures revealed *Edwardsiella tarda*, *Escherichia coli* and *Streptococcus anginosus*. Blood cultures yielded *Streptococcus anginosus*. Of note, *E. tarda* was susceptible to cefoxitin, 3rd and 4th generation cephalosporins, beta-lactam/beta-lactamase inhibitor combinations, aztreonam, carbapenems, aminoglycosides, trimethoprim-sulfamethoxazole, levofloxacin (MIC 0.5 µg/ml), and tigecycline. It was resistant to tetracycline. Antibiotics were changed to intravenous ceftriaxone and metronidazole. On day 2, his leukocytosis initially increased from 5.2 K/µL to 20.9 K/µL, but improved down to 8.5 K/uL by day 8. The patient remained afebrile and hemodynamically stable and was discharged from the hospital 8 days after admission to complete a total 14 day course of antibiotics with intravenous ceftriaxone and oral metronidazole. CT abdomen performed 3 weeks later off antibiotics demonstrated resolution of the perihepatic abscess. Four months later, the patient returned to the hospital with recurrence of RUQ abdominal pain but without clinical signs of sepsis. CT abdomen showed acute cholecystitis and new perihepatic collections, which were percutaneously drained. Fluid cultures grew *Escherichia coli* and *Enterococcus faecium.* Blood cultures were negative. There was no growth of *E. tarda,* indicating that *E. tarda* was successfully eradicated by prior antibiotic therapy.

## Discussion

*Edwardsiella tarda* is a motile, gram negative, facultative anaerobe, and a member of the *Enterobacteriales* family [1]. It is typically isolated in freshwater and marine environments and in animals that inhabit these environments. The colonization rate of *E. tarda* in humans ranges from 0.007% among Japanese to 1.0% among Panamanians [1]. Contact with infected animals and the consumption of contaminated foods are risk factors for acquisition of *E. tarda*. Although gastroenteritis is the most common manifestation of *E. tarda*, extraintestinal diseases, such as bacteremia, meningitis, cholecystitis, and peritonitis have been reported [1], [2], [3], [4], [5], [6], [7], [8].

Although *E. tarda* is a rare human pathogen, there is a higher rate of extraintestinal infection in immunocompromised patients and in subacute and chronic diseases. Our patient had several underlying systemic conditions and a history of hepatobiliary disease, which are reported risk factors for *E. tarda* infection [1], [3], [4]. In immunocompromised individuals, sepsis caused by *E. tarda* has a reported mortality of approximately 50% [1]. In our patient, CT imaging revealed cholelithiasis, which possibly resulted in the bilioenteric fistula, and eventually, cholecystitis, perforation, and perihepatic abscess. These findings, along with our patient’s history of chronic cholecystitis and slow clinical onset of symptoms, indicate either a subacute or chronic disease process, which increases the opportunity for facultative anaerobes to grow.

Anaerobes are also more commonly isolated in chronic inflammation compared to acute inflammatory diseases [11]. It is postulated that aerobic bacteria can lower the oxygen tension and pH in the acute stages of polymicrobial infection, which allows for the predominance of anaerobes in the chronic stages of illness [10]. Chronic biliary infections also tend to be polymicrobial, which are more pathogenic than single organism infections [10]. This is thought to be due to synergy between anaerobic and aerobic (both facultative and obligate) bacteria [9], [12]. In an in vivo abscess model in mice examining the synergistic effect between aerobes and anaerobes, the number of aerobic and facultative bacteria increased many more times compared their anaerobic counterparts, suggesting that the virulence of aerobes may be increased in the presence of anaerobes [9]. Some of these mechanisms include the ability of anaerobes to inhibit phagocytosis of aerobes by leukocytes, the production of essential growth factors, and the lowering of oxidation-reduction potentials in host tissues [9], [12].

In our patient, only *Streptococcus anginosus* was isolated in the blood, while the biliary abscess contained *S. anginosus, E. coli, and E. tarda*. Although *Streptococcus anginosus,* a facultative anaerobe, is part of the normal commensal flora of human mucous membranes, it has been known to cause invasive pyogenic infections and bacteremia [15], [16]. *S. anginosus* has also demonstrated increased pathogenicity in abscess formation in the presence of anaerobes in odontal and pulmonary infections, suggesting a possible synergistic effect with anaerobic bacteria [13], [14]. It is possible that there may be a synergistic relationship among the pathogens in this patient, resulting in increased virulence of *S. anginosis,* which was found in both the bloodstream and in the abscess cultures. More studies would be useful to further investigate this.

While there are case reports of *E. tarda* alone causing serious extraintestinal diseases, the role of *E. tarda* in the pathogenesis of this mixed infection is not clear. *E. tarda* is not a normal human enteric bacteria, but it has been isolated from the stool of patients without gastroenteritis [2], [3]. Whether *E. tarda* is a transient or long-term colonizer of the gastrointestinal tract is unknown. A recent *E. tarda* induced transcriptome profile study revealed numerous expressed immune genes including receptor genes, cytokines interferon-regulated genes that were associated with infection. The authors concluded that these newly expressed genes were induced by virulence determinants present in *E. tarda*. The expression of such genes could allow the organisms to evade the immune system which could lead to either transient or long-term colonization of the gastrointestinal tract. There have been no follow-up human findings to determine if *E. tarda* persists in stool after clinical resolution. While the isolation of *Streptococcus anginosis* and/or *E. coli* most likely contributed to the pathogenesis of the abscess in our case, the accompanying identification of *E. tarda* and its contribution to abscess formation remains unclear. In addition, more studies are needed to investigate the relationship between *E. tarda* and the microbiome and its possible role in persistence.

## Funding

This research did not receive any specific grant from funding agencies in the public, commercial, or not-for-profit sectors.

## CRediT authorship contribution statement

**Kimberly Pham** Participated in the initial writing of the manuscript**, Yuexiu Wu**, Participated in the initial writing of the manuscript**, Glenn Turett**, Participated in the review and editing of the manuscript**, Nishant Prasad**, Participated in the review and editing of the manuscript**, Lok Yung**, Participated in the review and editing of the manuscript**, George D. Rodriguez**, Participated in the review and editing of the manuscript**, Sorana Segal-Maurer**, Participated in the review and editing of the manuscript**, Carl Urban**, Participated in the review and editing of the manuscript**, James Yoon**, Participated in the diagnostic process, writing, review and editing of the manuscript.

## Consent

Consent to publish was not obtained since the case report does not contain any personal identifiers.

## Ethical approval

All authors have agreed for authorship, read and approved the manuscript, and given consent for publication of the manuscript.

## Declaration of Competing Interest

All authors report no potential conflicts of interest.
